# Environmental heterogeneity shapes the C and S cycling-associated microbial community in Haima's cold seeps

**DOI:** 10.3389/fmicb.2023.1199853

**Published:** 2023-07-04

**Authors:** Yu Chen, Tianjiao Dai, Niu Li, Qiqi Li, Yuanjiao Lyu, Pengfei Di, Lina Lyu, Si Zhang, Jie Li

**Affiliations:** ^1^Southern Marine Science and Engineering Guangdong Laboratory (Guangzhou), Guangzhou, China; ^2^CAS Key Laboratory of Tropical Marine Bio-resources and Ecology, South China Sea Institute of Oceanology, Chinese Academy of Sciences, Guangzhou, Guangdong, China; ^3^School of Water Resources and Environment, China University of Geosciences (Beijing), Beijing, China

**Keywords:** environmental heterogeneity, faunal aggregates, cold seep, microbiome, C and S cycles

## Abstract

Environmental heterogeneity in cold seeps is usually reflected by different faunal aggregates. The sediment microbiome, especially the geochemical cycling-associated communities, sustains the ecosystem through chemosynthesis. To date, few studies have paid attention to the structuring and functioning of geochemical cycling-associated communities relating to environmental heterogeneity in different faunal aggregates of cold seeps. In this study, we profiled the microbial community of four faunal aggregates in the Haima cold seep, South China Sea. Through a combination of geochemical and meta-omics approaches, we have found that geochemical variables, such as sulfate and calcium, exhibited a significant variation between different aggregates, indicating changes in the methane flux. Anaerobic methanotrophic archaea (ANME), sulfate-reducing, and sulfide-oxidizing bacteria (SRB and SOB) dominated the microbial community but varied in composition among the four aggregates. The diversity of archaea and bacteria exhibited a strong correlation between sulfate, calcium, and silicate. Interspecies co-exclusion inferred by molecular ecological network analysis increased from non-seep to clam aggregates and peaked at the mussel aggregate. The networked geochemical cycling-associated species showed an obvious aggregate-specific distribution pattern. Notably, hydrocarbon oxidation and sulfate reduction by ANME and SRB produced carbonate and sulfide, driving the alkalization of the sediment environment, which may impact the microbial communities. Collectively, these results highlighted that geofluid and microbial metabolism together resulted in environmental heterogeneity, which shaped the C and S cycling-associated microbial community.

## Introduction

Cold seeps are the leakage of fluids rich in methane and other hydrocarbons to the seafloor at specific sites at the continental margins (Paull et al., 1984; Orcutt et al., 2010), sustaining some of the richest ecosystems in the deep sea. Thriving faunal aggregates are usually found in cold seeps, making them signs for the oases in the deep sea. However, the composition of biota appears to vary between aggregates. For example, Vesicomyidae clams, mussels, and tubeworms seldom occur in one aggregate. On the one hand, the accessibility of resources for the symbiotic chemoautotrophs they depend on, such as methane and sulfide, determines their distribution patterns (Jørgensen and Boetius, 2007), indicating heterogeneous environments between aggregates. However, activities of animals, such as the up-and-down movement of Vesicomyidae clams in the sediment, can change the sediment environment, creating new niches for other organisms (Cordes et al., 2005; Bertics et al., 2007; Fischer et al., 2012), which is termed “niche construction” (Laland et al., 2016). Thus, environmental heterogeneity is usually reflected by different faunal aggregates in cold seeps, which are a result of a combination of geological and biological effects. Sediment microbes, especially the chemosynthetic species, serve the cold seep fauna with energy and resources, which act as the keystones for cold seep ecosystems. Although a great number of surveys have revealed the diverse microbial lineages inhabiting the cold seep sediment (Levin, 2005; Ruff et al., 2015), there is still a lack of holistic understanding of the structuring of microbial communities and their functionality in different faunal aggregates.

In recent decades, studies on the colonization and development of cold seep microbiota have been limited by the knowledge of cold seep development as well as deep-sea survey approaches. Recently, a study at Håkon Mosby Mud Volcano found that the microbial community showed clear alternation with increasing distance from the eruption center, representing a time-series succession (Ruff et al., 2019). The sulfur oxidizers and reducers colonized the sediment prior to the ANMEs (Ruff et al., 2019). Furthermore, the sulfur-utilizing bacteria were found to colonize prior to the methanotrophs in the Cinder Cones methane seep (Thurber et al., 2020). Clues from a fluid-ceased hydrothermal vent chimney demonstrated that the sediment microbiome experienced a significant transition in both community structure and energy-yielding metabolic potentials, which is probably driven by the availability of different energy sources at different stages (Hou et al., 2020). For example, the *Nitrospirae* clade played an essential role in sulfide oxidation, making a significant contribution to environmental changes once the fluid ceased, ultimately driving the succession of the community (Hou et al., 2020) and indicating that nutrition and energy sources fundamentally influenced the structure of the microbial community. Therefore, the environment of the deep-sea ecosystem significantly impacted the geochemical cycling associated with communities. Conversely, microbial metabolic cascades are probably able to reshape the environment and create new niches (Laland et al., 2016; Hou et al., 2020), which means that environmental heterogeneity and microbial community structuring and functioning are clearly correlated in deep-sea ecosystems. However, neither the keystone lineages of the geochemical cycling-associated communities nor their function in different faunal aggregates in cold seeps have been clarified to date.

Cold seep ecosystems are sustained by the chemosynthesis of microorganisms related to carbon and sulfur cycling. For example, microbial degradation of hydrocarbons in cold seeps is usually coupled with sulfate reduction (Kleindienst, 2012), while inorganic carbon fixation could be driven by sulfide oxidation (Chakraborty et al., 2020). Supplementation of carbon (i.e., methane and hydrocarbons) and sulfur compounds (i.e., sulfate and sulfide) fundamentally impacts the metabolic patterns of microorganisms (Dong et al., 2020; Joye, 2020), implying the various geochemical cycling-associated communities in different faunal aggregates. However, the influence of environmental heterogeneity on the structuring and functioning of C and S cycling-associated microbial communities remains unclarified in cold seeps. In this study, we hypothesized that sedimental environments in different faunal aggregates play a key role in the structuring and functionality of C and S-cycling-associated microbial communities. Thus, we elucidated the composition, diversity, interactions, and functional potential of the sediment microbiome of four faunal aggregates in Haima's cold seep, South China Sea (SCS), providing clear evidence for the role of environmental heterogeneity in structuring and functionality of the C and S cycling-associated microbial community.

## Materials and methods

### Sample collection

**Figure 1:**
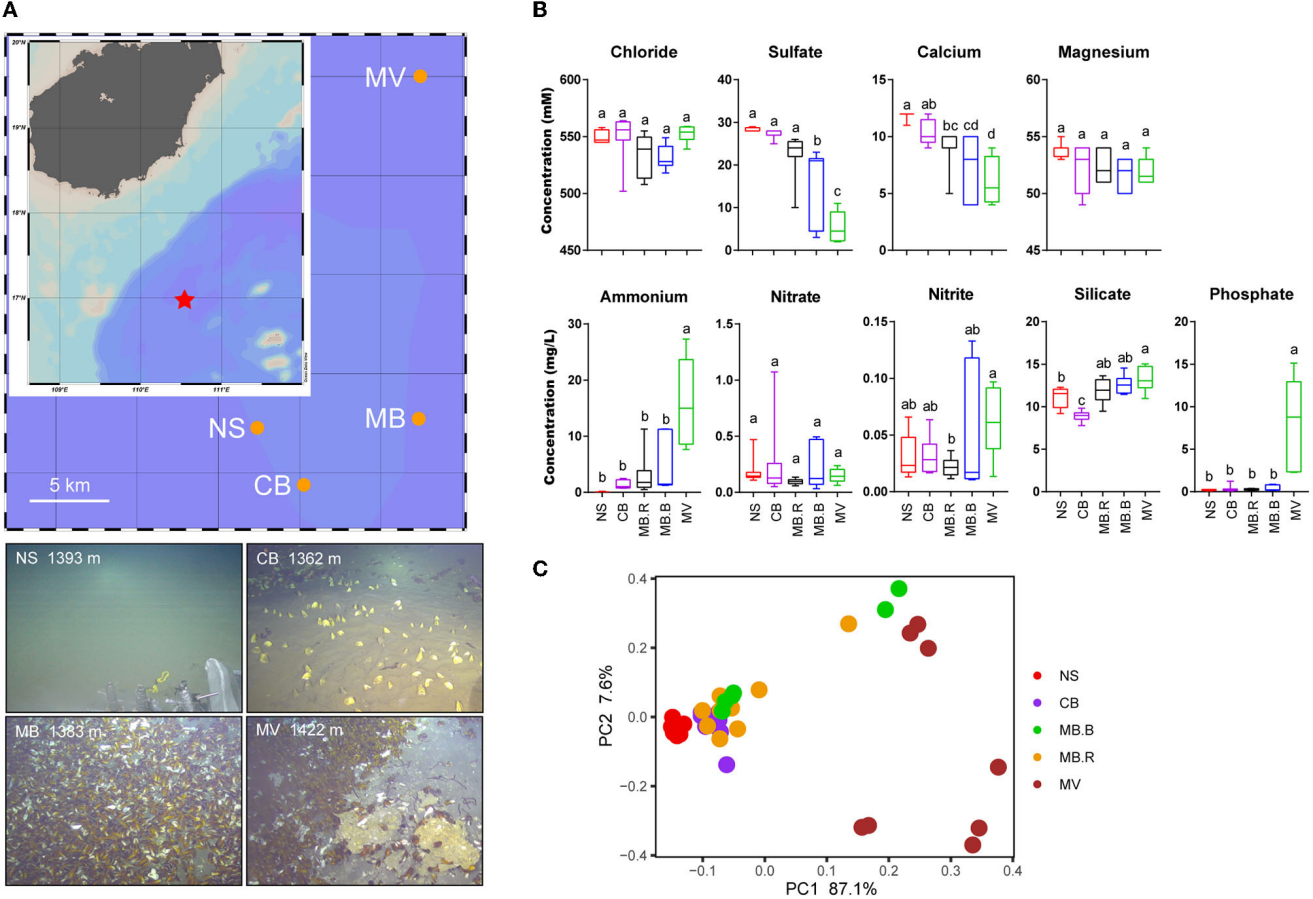
Location and environmental traits of different aggregates of Haima cold seep. **(A)** Haima cold seep is located to the southwest of Hainan Island, South China Sea*.. The landscape of four aggregates was illustrated in the images. **(B)** Concentrations of chloride, sulfate, calcium, magnesium, ammonium, nitrate, nitrite, silicate, and phosphate in different aggregates. Lowercase letters above the box plots indicated significant differences (ANOVA, *p* < 0.05). **(C)** Principal component analysis based on Euclidean dissimilarity showed a clear separation by samples of geochemical variables between aggregates.

Sediment push cores were obtained from four habitats of the Haima cold seep in the north of SCS (16.9°N, 110.4°E) via remotely operated vehicle (ROV) in September 2020. The non-seepage habitat (NS) was normal seafloor (1,393 m, Figure 1A), representing the status before seepage. Clam bed (CB) inhabited Vesicomyidae clams, and microbial mats (1,362 m, Figure 1A). MB and MV were both mussel habitats. MB possessed large mussel aggregates on muddy sediment (1,394 m, Figure 1A). Gas venting and large carbonate crusts were found in the dominant mussel MV (1,440 m, Figure 1A). One push core was retrieved from NS (25 cm), CB (35 cm), and MV (24 cm), respectively, and two push cores were obtained from MB (24 and 23 cm). Porewater was extracted using a syringe under vacuum upon retrieval at 4°C, preserved with a 10 μL saturated HgCl_2_ solution, and stored at 4°C under an N_2_ headspace. Then, the push cores were cut into 3 cm per sample and stored at −80°C before use. The methane concentration of the bottom seawater was recorded using a sensor on the Haima ROV. The maps were illustrated with Ocean Data View version 5.4.0 (Schlitzer, 2015).

### Geochemical analysis

Geochemical variables were analyzed following our previous study (Luo et al., 2015). Briefly, porewater samples were diluted 1:500 and 1:200 with ddH_2_O to determine the concentrations of negative ions [sulfate (SO${}_{4}^{2-}$) and chloride (Cl^−^)] and positive ions [magnesium ion (Mg^2+^) and calcium ion (Ca^2+^)], using a Dionex ICS-900 ion chromatography. An Ion Pac AS23-type column and a 4.5 mM Na_2_CO_3_ and 0.8 mM NaHCO_3_ mixed solution in an anion system were used to determine SO${}_{4}^{2-}$ and Cl^−^ concentrations. An IonPac CS12A-type column and an 11 mM H_2_SO_4_ solution in a cation system were used to determine magnesium [Mg^2+^] and calcium [Ca^2+^] concentrations. Concentrations of nitrate [NO${}_{3}^{-}$], nitrite [NO${}_{2}^{-}$], ammonia [NH${}_{4}^{+}$], silicate [SiO${}_{3}^{2-}$], and phosphate [PO${}_{4}^{2-}$] were measured using a Lachat QuickChem8500 autoanalyzer (Lachat Instruments, Loveland, CO, USA) following standard colorimetric methods (Grasshoff et al., 2009).

### DNA extraction

Total DNA was extracted from 2 g of sediments using the TGuide S96 Magnetic Soil/Stool DNA Kit [Tiangen Biotech (Beijing) Co., Ltd.] for full-length 16S rRNA gene amplicon and metagenomic sequencing according to the manufacturer's instructions. The DNA concentration of the samples was measured with the Qubit dsDNA HS Assay Kit and Qubit 4.0 Fluorometer (Invitrogen, Thermo Fisher Scientific, Oregon, USA).

### Full-length 16S rRNA gene amplicon sequencing

The bacterial and archaeal 16S rRNA genes were amplified using the 27F/1492R primer pairs (Delong, 1992) and 20F/1492R primer pairs (Delong, 1992; Chen et al., 2016), respectively. PCR progress was shown as follows: 95°C for 2 min; followed by 25 cycles of 98°C for 10 s, 55°C for 30 s, and 72°C for 90 s; and finished with 72°C for 2 min. Amplicons were purified with Agencourt AMPure XP beads (Beckman Coulter, Indianapolis, IN). SMRTbell libraries were prepared with the SMRTbell Express Template Prep Kit 2.0 (Pacific Biosciences) and sequenced on the PacBio Sequel II platform. Raw data were primarily filtered and demultiplexed using the SMRT Link software (version 8.0) with minPasses ≥5 and minPredictedAccuracy ≥0.9 to obtain the circular consensus sequencing (CCS) reads. The LIMA (version 1.7.0) was employed to assign the CCS to the corresponding samples based on the barcodes. CCS reads without primers and with a length over the range of 1,200–1,650 bp were discarded, and quality filtering was performed using Cutadapt (Martin, 2011; version 2.7). Amplicon sequence variants (ASV) were generated with DADA2 (Callahan et al., 2016) in QIIME2 (Bolyen et al., 2019), and the ones with an abundance of < 0.005% were filtered (Bokulich et al., 2013). The ASV matrices were rarefied by the minimum sample size for archaea (17,388) and bacteria (6351). Taxonomy annotation was performed based on the Naive Bayes classifier in QIIME2 (Bolyen et al., 2019) using the SILVA database (Quast et al., 2012; release 132).

### Community diversity and phylogenic analysis

Alpha diversity indices were calculated with the picante (Kembel et al., 2010) package in R. Bray-Curtis Similarity Index-based non-metric multidimensional scaling (NMDS) was calculated via the vegan package (Dixon, 2003) in R. Analysis of similarity (ANOSIM), permutational multivariate analysis of variance (PERMANOVA), Mantel test, and Student's *t*-test were performed via vegan (Dixon, 2003) package (with 9999 permutations for ANOSIM, PERMANOVA, and Mantel test). Distance-based redundancy analysis was performed via a vegan package. Spearman correlation was calculated with the psych (Revelle, 2011) package in R. For phylogenic analysis, the sequence of 16S rRNA gene ASVs was aligned using the MUSCLE algorithm (Edgar, 2004). Maximum likelihood trees were constructed using the Tamura-Nei model with all sites, bootstrapped with 500 replicates by MegaX (Kumar et al., 2018).

### Molecular ecological network analysis (MENA)

To identify the interactions of microbial species at different aggregates, molecular ecological networks (MENs) were constructed on the basis of the Pearson correlations of bacterial and archaeal ASV abundances, followed by an RMT-based approach that determines the correlation cut-off threshold in an automatic fashion (Luo et al., 2006, 2007; Zhou et al., 2010) with the Molecular Ecological Network Analyses Pipeline (Deng et al., 2012). Archaeal and bacterial ASVs from the same sample were pooled for the construction of MENs (6–9 samples for each man, Supplementary Table 1), and only the ASVs present in at least 6 samples were included for correlation calculation (Deng et al., 2012).

The topological indices, including total nodes, total links, power-law fitting of node degrees, average K (avgK), average CC (avgCC), average path distance (geodesic distance, GD), geodesic efficiency (E), modularity, harmonic geodesic distance (HD), centralization of stress centrality (CS), centralization of betweenness (CB), centralization of eigenvector centrality (CE), density, modularity, and efficiency, were calculated in the MENAP interface (Deng et al., 2012). Random networks were generated for each empirical network by randomly rewiring the links among the nodes while constraining nodes and links, following the Maslov–Sneppen procedure (Maslov and Sneppen, 2002) in the MENAP.

### Metagenomic analysis

A total of 10 nanograms of DNA extracted from 2 g of sediments using the TGuide S96 Magnetic Soil/Stool DNA Kit [Tiangen Biotech (Beijing) Co., Ltd.] was used to produce a library via the VAHTS^®^ Universal Plus DNA Library Pren Kit for Illumina, and the pooled libraries were sequenced on the Illumina Novaseq 6000 platform. Raw data were primarily filtered using Trimmomatic (Bolger et al., 2014; version 0.33) with the following parameters: Pe leading 3, trailing 3, sliding window 50:20, and minLen 120. Filtered reads were assembled via MEGAHIT (Li et al., 2015; version v1.1.2) for each sample, and short contigs (< 300 bp) were filtered. Identification of the opening reading frame (ORF) was conducted using MetaGeneMark (http://exon.gatech.edu/meta_gmhmmp.cgi, version 3.26) with the default parameter. Non-redundant genes were clustered in line with a sequence similarity of 95% and a coverage of 90% using MMseq2 (https://github.com/soedinglab/mmseqs2, version 11-e1a1c). Non-redundant genes were annotated with the Kyoto Encyclopedia of Genes and Genomes (KEGG), Swiss-Prot, and NR databases in DIAMOND (version 0.9.29, cutoff: e-value 1e-5). To construct the metabolic pathway, genes with an average relative abundance of over 1e-06 were mapped via the Reconstruct tool in KEGG (Kanehisa et al., 2021). The schematic diagram was generated via BioRender (https://app.biorender.com) and modified manually.

## Results

### Geochemical variables for different aggregates

A total of four aggregates, namely, non-seepage muddy seafloor (NS), clam bed (CB), mussel bed (MB), and mussel aggregates with methane vent and carbonate rocks (MV), were found in Haima's cold seep during a cruise in 2020. Dead clams were found in the mussel habitats (MB and MV), but mussels (either living individuals or remnants) were not found in CB (Figure 1A). Accordingly, based on our observations and previous studies (Bergquist et al., 2003; Cordes et al., 2006; Bowden et al., 2013), the aggregates may represent different stages of cold seep development. Geochemical variables in the sediment showed evident differences among aggregates (Figure 1B, Supplementary Figure 1). Sulfate and calcium concentrations showed dramatic vertical declines in the push cores obtained from MB and MV (Supplementary Figure 1). The methane concentrations for bottom seawater were recorded as 0, 1.1, 5.6, and over 9.9 μmol for NS, CB, MB, and MV, respectively, indicating an increase in methane flux from NS to MV. Ammonium and phosphate, usually derived by methanogenesis (Quevedo et al., 1996; Lippens and De Vrieze, 2019), increased in concentration from NS to MV. The decreased concentrations of sulfate and calcium from NS to MV indicated an increase in AOM intensity. The concentration of silicate increased from CB to MV, indicating a more alkaline environment that promoted its solvation (Van Cappellen and Qiu, 1997; Smrzka et al., 2015). Variation of geochemical variables was not found in a vertical direction, except in MB. The dramatic decline of sulfate in the subsurface samples indicated that they were in the sulfate methane transition zone (SMTZ). The geochemical metadata exhibited a significant difference between aggregates and an increase in heterogeneity in MB.B and MV (ANOSIM, *R* = 0.61, *p* < 0.001, Figure 1C). Therefore, we divided the sediment samples into five groups (NS, CB, MB.R, MB.B, and MV) according to the geochemical variables (Supplementary Table 1).

### Microbial community diversity and composition

**Figure 2:**
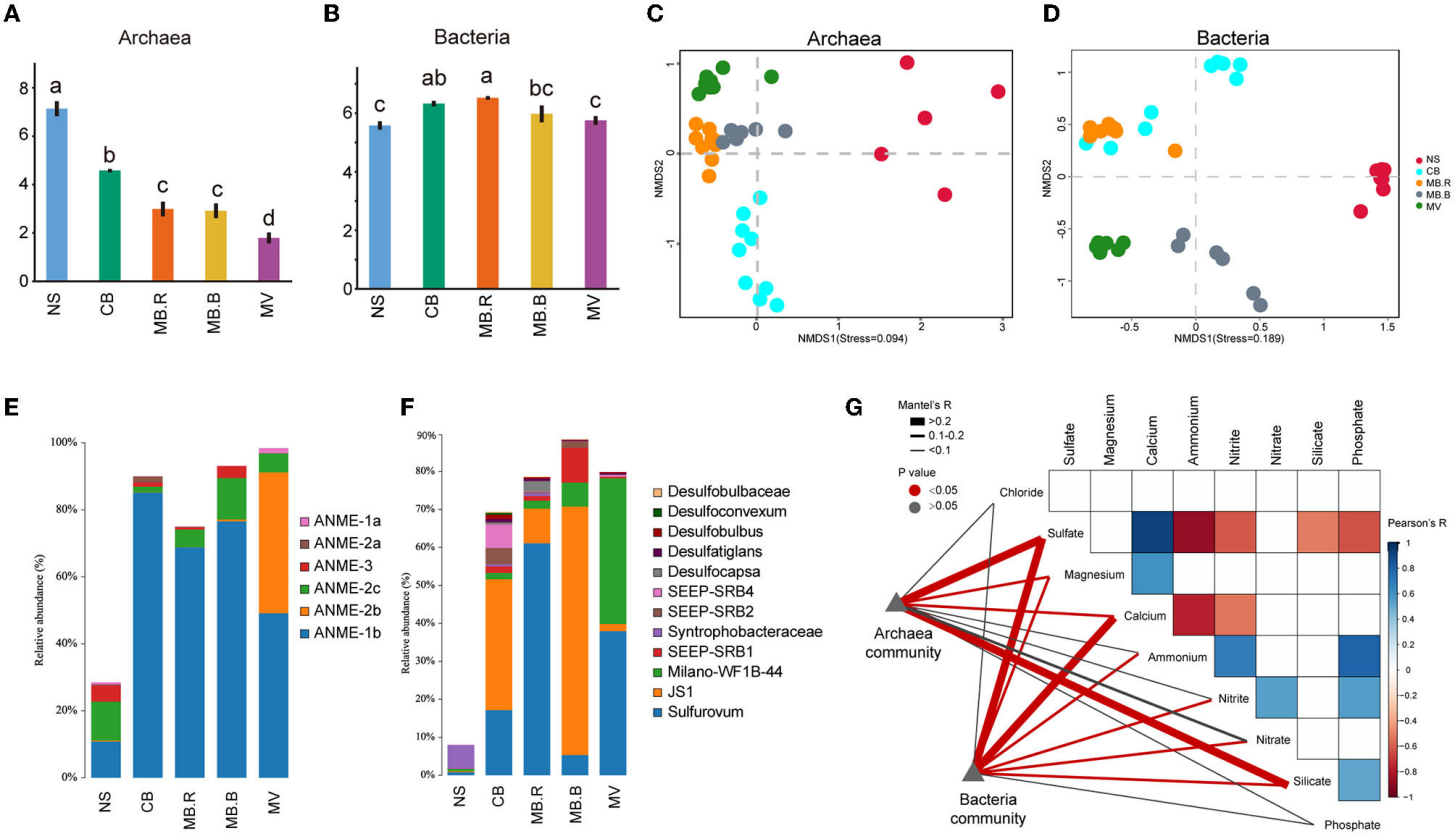
Microbial diversity in different aggregates of Haima cold seep. **(A, B)** The Shannon index of archaeal and bacterial communities. Different letters indicated significant differences between aggregates (one-way ANOVA, *p* < 0.05 adjusted with Tukey). **(C, D)** Non-metric multidimensional scaling analysis (NMDS) based on Bray-Curtis dissimilarity for archaeal **(C)** and bacterial **(D)** communities. **(E, F)** Composition of archaeal and bacterial communities. The vertical axis indicated the relative abundance of ANME in the total archaea community **(E)** and SRB, SOB, JS-1, etc., in the total bacteria community **(F)**. **(G)** The Pearson correlation of geochemical variables and their relationship with the composition of archaeal and bacterial communities was evaluated using the Mantel test. The colored boxes indicated a strong correlation of geochemical variables (Pearson, |*R*| > 0.5, *p* < 0.05).

A total of 1,099,280 full-length bacterial 16S rRNA gene sequences from 40 samples and 676,132 archaeal sequences from 38 samples were retrieved (Supplementary Table 1). The Shannon index of the archaeal community decreased from NS to MV (Figure 2A), while that for the bacterial community increased from NS to MB but decreased at MV (Figure 2B). Non-metric multidimensional scaling (NMDS) based on Bray–Curtis distance revealed a clear alternation in archaeal and bacterial community structure in different aggregates of Haima's cold seep (Figures 2C, D), which was also supported by PERMANOVA and ANOSIM (Supplementary Tables 3, 4). Anaerobic methanotrophic archaea (ANME) affiliated with Euryarchaeota were the predominant archaea in CB, MB, and MV, comprising 81.3–98.4% of the archaeal community. The ANME-2b clade was hardly found in NS, MB, and MV but dominant in MV (Figure 2E). For bacterial communities, the abundance of *Proteobacteria, Atribacteria*, and *Campylobacterota* increased from 28.7% in NS up to 72.7–89.6% in CB, MB, and MV. *Proteobacteria* were the most diverse bacterial phylum and mainly consisted of sulfate-reducing bacteria (SRB). *Syntrophobacteraceae* were dominant and prevalent in NS, while SEEP-SRB1, SEEP-SRB2, SEEP-SRB4, *Desulfocapsa*, and *Desulfatiglans* were dominant in CB and MB. Milano-WF1B-44 became the predominant *Proteobacteria* in MV, whose relative abundance rose to 38.4%. JS-1 clade affiliated with Atribacteria largely inhabited CB and MB, comprising 9.1–65.5% of bacteria. The sulfide-oxidating bacteria (SOB) *Sulfurovum* (Assié et al., 2020), affiliated with Campylobacterota, has been abundant since CB, comprising 5.3–61.0% of bacteria (Figure 2F).

The community diversity and its correlation with environmental variables were evaluated for the four aggregates. Alpha diversity indices of the archaeal community were largely correlated with concentrations of sulfate, calcium, ammonium, and silicate (Spearman, |*R*| > 0.3, *p* < 0.05, Supplementary Table 5). However, the diversity indices of the bacterial community were correlated with concentrations of nitrite and nitrate (Spearman, |*R*| > 0.3, *p* < 0.05, Supplementary Table 6). The composition of the archaeal community was strongly correlated with concentrations of sulfate and silicate (Mantel test, *R* > 0.2, *p* < 0.05) and exhibited a weak but significant correlation with magnesium (Mantel test, 0.1 < *R* < 0.2, *p* < 0.05). In contrast, the composition of the bacterial community was strongly correlated with concentrations of sulfate and calcium (Mantel test, *R* > 0.2, *p* < 0.05) and exhibited a weak but significant correlation with concentrations of magnesium, ammonium, nitrate, and nitrite (Mantel test, 0.1 < *R* < 0.2, *p* < 0.05, Figure 2G).

### Network associations

**Figure 3:**
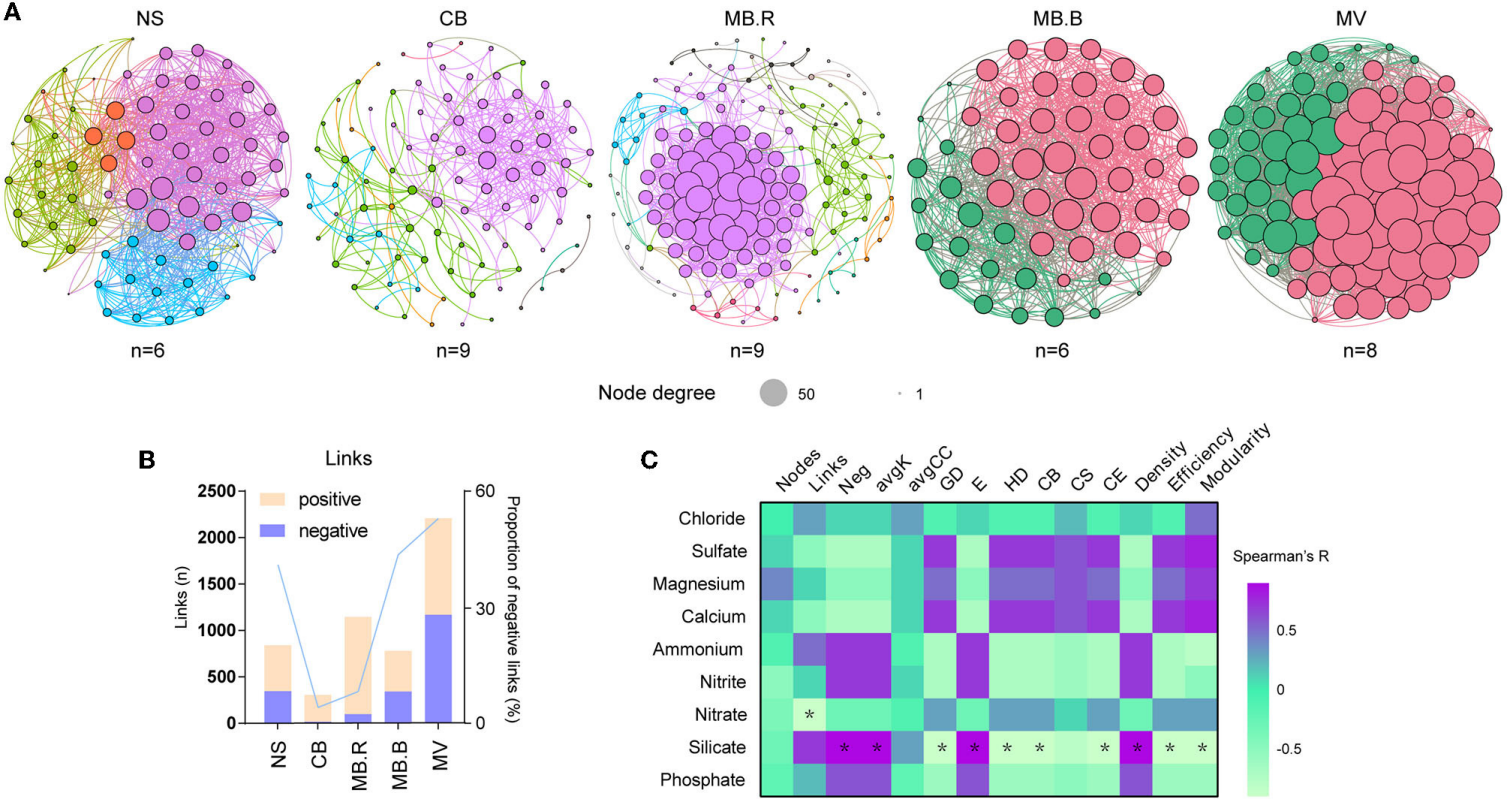
Variation of microbial networks over succession. **(A)** Molecular ecological networks (MENs) constructed with archaeal and bacterial ASVs for NS to MV. The nodes were dyed by module. The size of the nodes indicated the node degree. **(B)** The number of links and proportion of negative links in the network for different aggregates. **(C)** The Spearman correlation of network topological indices and geochemical variables. Asterisks indicated strong correlations (|*R*| > 0.8, *p* < 0.05). The sample number used for MEN construction was marked below the corresponding network. Neg, the proportion of negative links; avgK, average degree; GD, average path distance; E, geodesic efficiency; HD, harmonic geodesic distance; CB, centralization of betweenness; CS, centralization of stress centrality; CE, centralization of eigenvector centrality.

To evaluate the associations among microbial species, molecular ecological networks (MENs) were constructed using a random matrix theory (RMT)-based approach (Luo et al., 2006, 2007; Zhou et al., 2010). The network nodes represent microbial species, and links between nodes represent their abundances that were significantly correlated. All the networks displayed scale-free, small-world, and modular properties. They exhibited significant differences in topological indices from the corresponding random networks (Supplementary Table 7), suggesting that the constructed networks were ecologically meaningful. We found the number of network nodes and modules that peaked in MB.R (Figure 3A, Supplementary Table 7), which was in line with changes in bacterial species diversity (Figure 2D). However, the network nodes were most densely correlated in MV, indicating that the species were highly associated in this aggregate. The proportion of negative links was the lowest at CB but gradually increased to 52.8% in MV (Figure 3B). Such increases in negative link proportion were also found among ANME and SRB species, as well as between ANME, SRB, JS-1, and *Sulfurovum* species (Supplementary Figure 2). Moreover, topological indices of the networks, including the proportion of negative links (Neg), average degree (avgK), geodesic efficiency (E), and density, were positively correlated with silicate, while modularity, average path distance (GD), harmonic geodesic distance (HD), centralization of betweenness (CB), centralization of eigenvector centrality (CE), and efficiency were negatively correlated with silicate (Spearman, |*R*| > 0.8, *p* < 0.05, Figure 3C).

### Aggregate-specific distribution of carbon and sulfur cycles associated species

**Figure 4:**
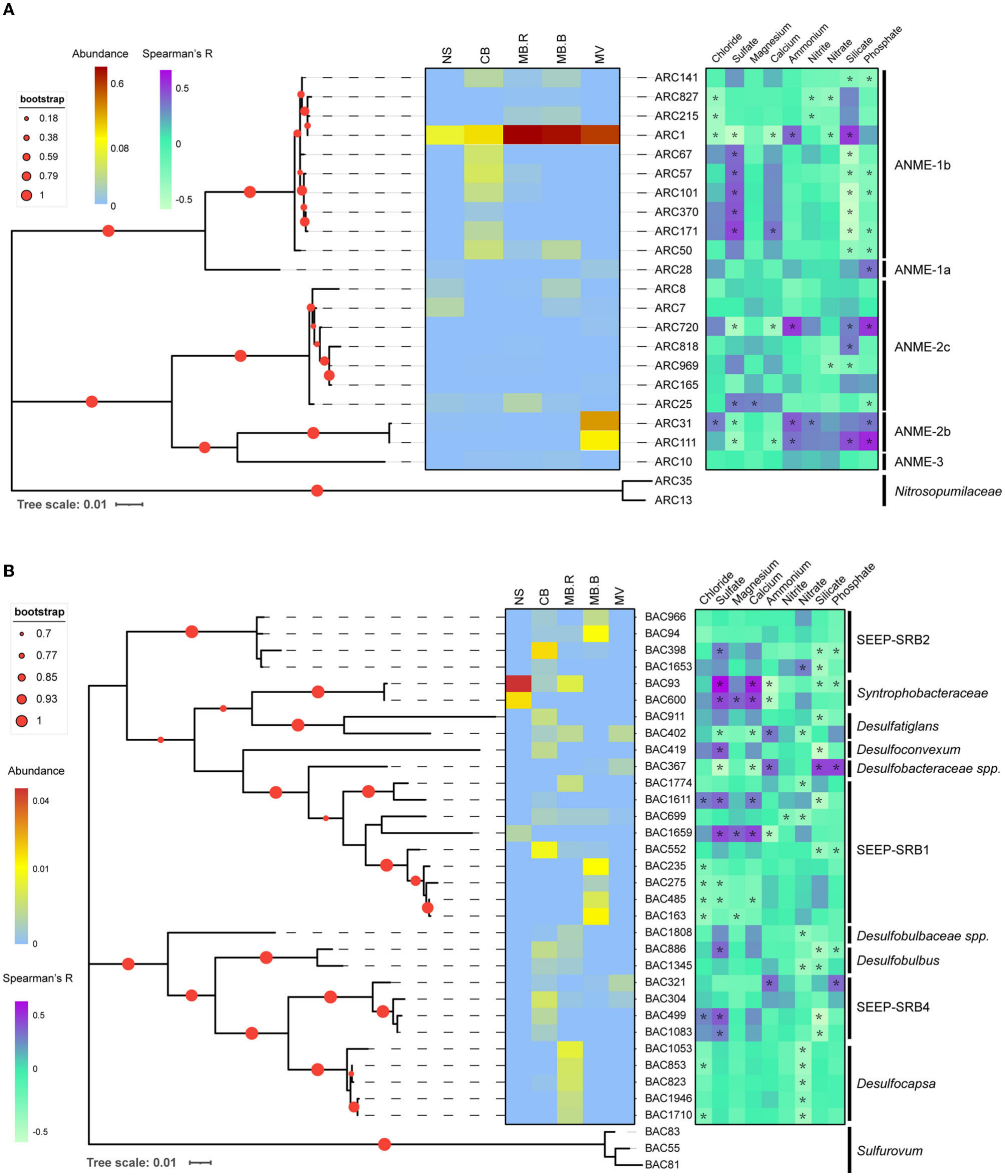
Phylogenic relationship and distribution pattern of ANME and SRB. Maximum likelihood phylogenic trees for networked ANME **(A)** and SRB **(B)** with 16S rRNA gene sequences. Sequences in *Nitrosopumilaceae* were selected as an outgroup for ANME and *Sulfurovum* for SRB. Asterisks indicated significant correlations between ASV abundance and geochemical variables (Spearman, |*R*| > 0.3, *p* < 0.05).

To evaluate the distribution patterns and their correlations with geochemical variables, phylogenetic trees were constructed for networked species affiliated with ANME, SRB, JS-1, *Sulfurovum*, and Milano-WF1B-44. ANME-1b was the dominant networked archaea, and most of them inhabited CB. Interestingly, the abundance of ARC1 kept increasing and peaked at MB. ARC31 and ARC111, affiliated with ANME-2b, were prevalent in MV and one of the dominant species. Other ANME species did not show an apparent aggregate-specific distribution pattern. The distribution of ANME species was largely positively correlated with sulfate (Spearman, *R* > 0.3, *p* < 0.05) but negatively correlated with silicate (Spearman, *R* < −0.3, *p* < 0.05). In contrast, ARC1, ARC31, and ARC111 have shown a negative correlation with sulfate and calcium (Spearman, *R* < −0.3, *p* < 0.05) but a positive correlation with silicate and phosphate (Spearman, *R* > 0.3, *p* < 0.05), indicating their prevalence in methane and correlation with AOM (Figure 4A). The networked JS-1 species, ubiquitous cold seep taxa involved in hydrocarbon fermentation (Liu et al., 2019; Dong et al., 2020), were specific in the CB and MB. Their distribution was dominantly correlated with sulfate and silicate (Spearman, |*R*| > 0.3, *p* < 0.05), indicating their sensitivity to the geochemical changes caused by AOM (Supplementary Figure 3).

SRB directly participates in the biogeochemical cycle of carbon and sulfur (Fukui et al., 1999; Kleindienst et al., 2014; Jaekel et al., 2015; Davidova et al., 2019), showing a clear aggregate-specific distribution pattern in Haima's cold seep. *Syntrophobacteraceae*, mediating the transformation of long-chain alkane to carbon dioxide or acetate (Tan et al., 2014a,b), were the dominant SRB in NS but were absent in MB.B and MV. SEEP-SRB1 and SEEP-SRB2, the well-known ANME partners (Kleindienst et al., 2012), were prevalent in CB and MB. Other networked SRB species, such as *Desulfocapsa, Desulfatiglans, Desulfoconvexum*, and *Desulfobulbus*, were candidate hydrocarbon degradaters (Lloyd et al., 2006; Bombach et al., 2009; Abu Laban et al., 2015; Kleindienst and Knittel, 2020). *Desulfocapsa* was specific to MB.R, while the others were prevalent in CB and MB. The distribution of SRB showed a clear species-specific pattern in correlation with geochemical variables, indicating the diversity of SRB taxonomy and its correlation with the environment (Figure 4B). *Sulfurovum* was manually divided into three clusters based on the phylogenetic relationships (Supplementary Figure 4). Clusters 1 and 3 were largely specific to MB.R, while Cluster 2 was widely distributed from CB to MV. Clusters 1 and 3 were negatively correlated with nitrate (Spearman, *R* < −0.3, *p* < 0.05). Cluster 2 negatively correlated with sulfate and calcium (Spearman, *R* < −0.3, *p* < 0.05) but positively correlated with ammonium, silicate, and phosphate (Spearman, *R* > 0.3, *p* < 0.05). Milano-WF1B-44 species were prevalent in MV and also showed a negative correlation with sulfate and calcium (Spearman, *R* < −0.3, *p* < 0.05) but a positive correlation with ammonium, silicate, and phosphate (Spearman, *R* > 0.3, *p* < 0.05, Supplementary Figure 5).

### Carbon and sulfur metabolic pathways

**Figure 5:**
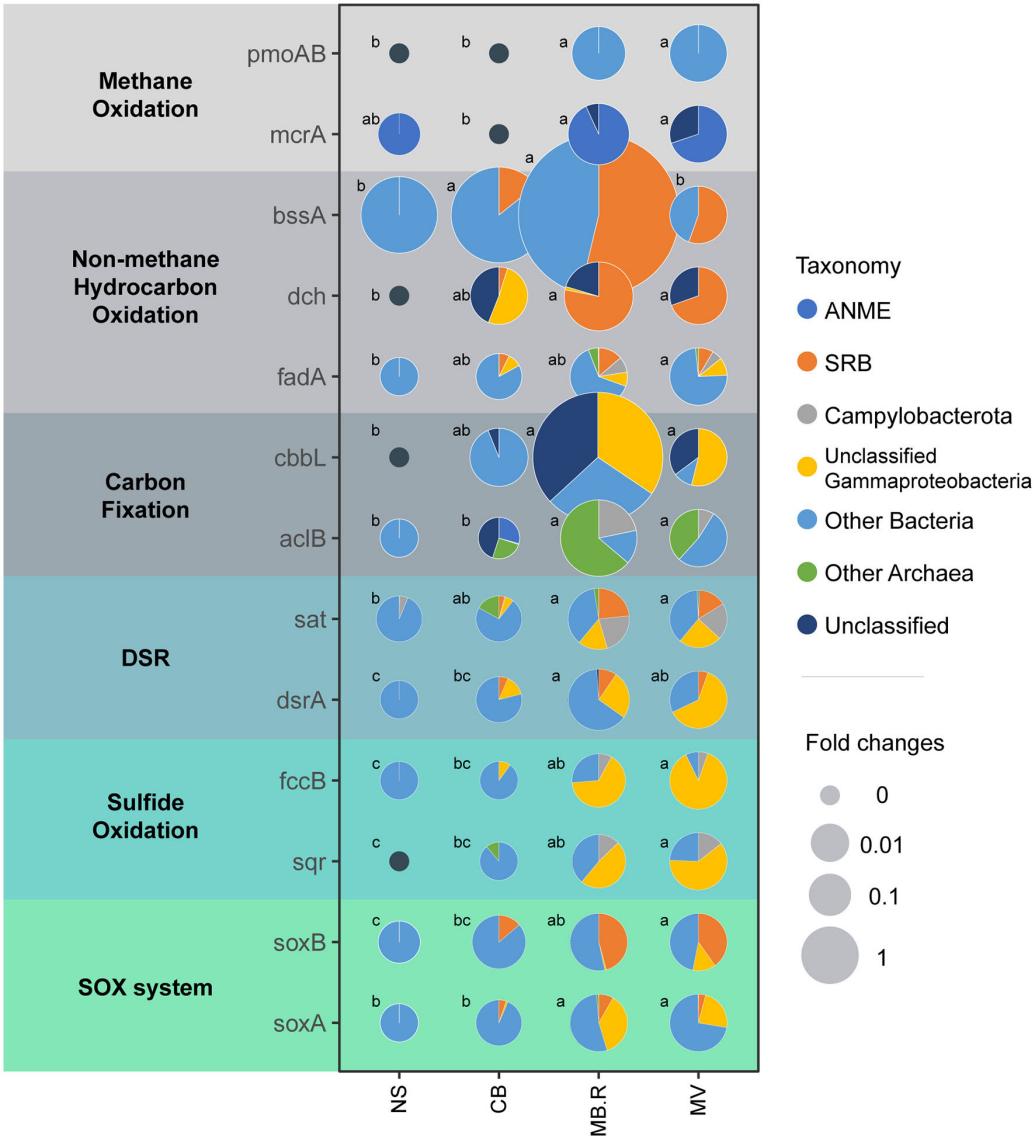
Abundance and taxonomic composition of key genes involved in carbon and sulfur metabolisms between different aggregates. Key genes involved in methane oxidation, non-methane hydrocarbon oxidation, carbon fixation, dissimilatory sulfate reduction (DSR), sulfide oxidation, and the SOX system were examined. The pie graphs illustrate the taxonomic composition of each gene in each aggregate. The relative abundance of each gene was normalized with MV as one, and the size of the pies indicated the fold changes. Different letters indicated a significant difference in abundance between aggregates (one-way ANOVA, *p* < 0.05 adjusted with Tukey).

To explore potential carbon and sulfur metabolic pathways and their changes in different aggregates, metagenomic approaches were applied to profile microbial functional genes (Supplementary Table 1). The *pmoA/B* genes encoding methane monooxygenase and the *mcrA* gene encoding methyl-coenzyme M reductase were prevalent in MB.R. and MV, but they were specific to bacteria or archaea, respectively, indicating the difference in methane metabolism pathways in these two kingdoms. The aromatic hydrocarbon biodegradation-related genes, *bssA* and *dch* (Boll et al., 2002; Winderl et al., 2007; Porter and Young, 2014), were prevalent in CB and MB.R and dominantly affiliated with SRB, indicating the deep involvement of SRB in aromatic hydrocarbon oxidation. The chain hydrocarbon oxidation-related *fadA* gene, encoding acyl-CoA dehydrogenase in β-oxidation pathway (Dong et al., 2020), was carried by diverse bacterial lineages and also prevalent in CB and MB.R. For carbon fixation, the *cbbL* gene encoding ribulose-bisphosphate carboxylase for the Calvin-Benson-Bassham (CBB) cycle and the *aclB* gene encoding ATP-citrate lyase of the reductive tricarboxylic acid (rTCA) cycle were both enriched in MB.R. The *cbbL* gene was carried out by some unclassified Gammaproteobacteria, implying the Milano-WF1B-44 clade. However, among the *aclB* gene carriers, we found Campylobacterota and archaea lineages. For sulfur metabolisms, genes encoding the essential enzymes in the dissimilatory sulfate reduction (DSR) pathway, *sat* and *dsrA*, were rare in NS but increased in abundance since CB, indicating an increase in DSR metabolism and peaked in MB.R. Other than the known SRB, lineages from Campylobacterota and Gammaproteobacteria were also supposed to carry these genes. The *sqr* and *fccB* genes encoding sulfide (quinone oxidoreductase and sulfide dehydrogenase for sulfide oxidation) exhibited a gradual increase from NS to MV. Campylobacterota and unclassified Gammaproteobacteria were carrying these genes, suggesting the potential involvement of *Sulfurovum* and Milano-WF1B-44 in sulfide oxidation in the cold seep. A similar pattern was also found in the SOX system-related genes, which were dominantly affiliated with SRB, unclassified Gammaproteobacteria, and other bacterial lineages (Figure 5).

**Figure 6:**
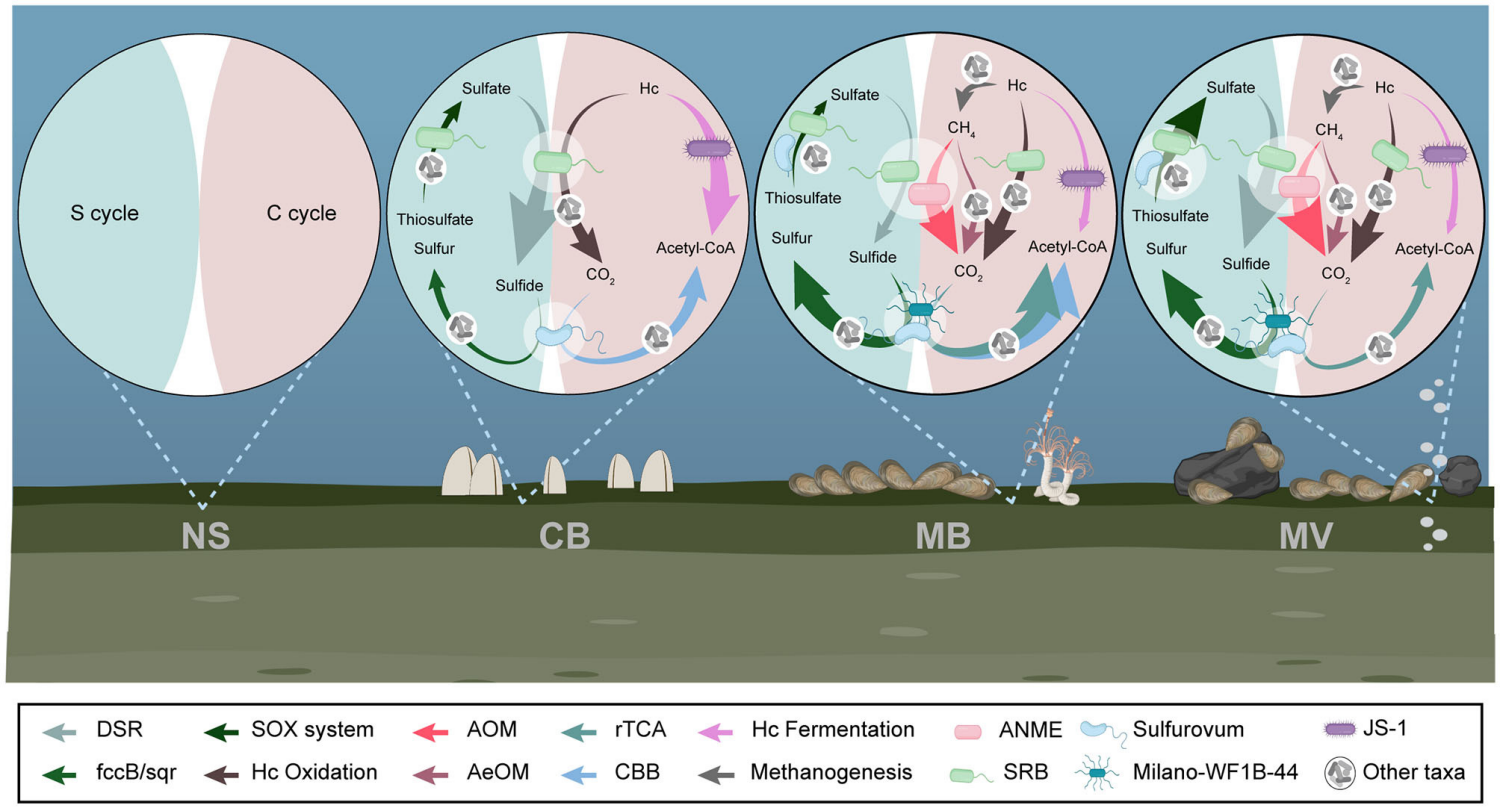
Carbon and sulfur metabolic pathways in different aggregates. Complete pathways for methane, hydrocarbon, and sulfur metabolisms were illustrated for each aggregate. DSR, dissimilatory sulfate reduction; AOM, anaerobic oxidation of methane; AeOM, aerobic oxidation of methane; rTCA, reductive tricarboxylic acid cycle; CBB, Calvin-Benson-Bassham cycle; ANME, anaerobic methanotroph; SRB, sulfate-reducing bacteria; Hc, hydrocarbon.

We constructed the potential carbon and sulfur metabolic pathways for each aggregate based on the annotated genes. Methane metabolic pathways, including aerobic and anaerobic oxidation of methane (AeOM and AOM), were identified in MB.R and MV. Complete benzoyl-CoA and β-oxidation pathways for aromatic and chain hydrocarbon degradation have been identified since CB, indicating that hydrocarbon degradation potentially could vary in intensity between different aggregates. The CBB cycle was only present in CB and MB for the carbon fixation mechanisms, while the rTCA cycle was specific to MB and MV, showing a noticeable preference for carbon fixation patterns. For the sulfur cycle, DSR, the SOX system, and the sulfide oxidation enzymes were all present since CB, indicating complete sulfur cycling in all aggregates but probably varying in metabolic intensity. None of the pathways mentioned above was identified in NS (Figure 6).

## Discussion

Haima cold seep is a typical hydrocarbon seep in which methane is the dominant component of its fluid (Guan et al., 2018). Increased methane flux was found in NS, MB, and MV, which was inferred based on methane, ammonium, and phosphate concentrations (Quevedo et al., 1996; Lippens and De Vrieze, 2019). This increased methane influx was accompanied by the upward movement of the SMTZ, as indicated by the depletion of sulfate, which even reached the sediment-water interface (Supplementary Figure 2). In most cold seep sediments, the AOM rate is far higher than the AeOM rate and usually positively correlated with the sulfate-reducing rate (Pop Ristova et al., 2012), making the sulfide-rich environment of cold seep sediments (Joye, 2020). In general, the pH of the sediment increases with methane flux in cold seeps as a result of the production of carbonate and sulfide by sulfate reduction coupled AOM (Kiel, 2010). Although pH or alkalinity was not measured in this study, we could infer an increase in pH by silicate concentration (Van Cappellen and Qiu, 1997; Smrzka et al., 2015), which is also supported by the previous finding that the alkalinity flux is lower in the clam aggregates than the mussel ones (Pop Ristova et al., 2012). Given that the faunal aggregates were also shaped by methane flux (Cordes et al., 2009; Bowden et al., 2013), we supposed that the methane flux fundamentally changed the environment (i.e., alkalization, carbon, and sulfur compounds) between different faunal aggregates, probably by impacting microbial communities and C, S cycling.

Microbes drove geochemical cycling and played a primary role as producers for cold seep ecosystems, but the structuring of the associated microbial community is still unclear to date. Increasing methane flux specifically benefits the methanotrophs, namely the ANMEs in this study, but is harmful to many other organisms. In addition, pH is beneficial to some archaeal lineages but harmful to others, which essentially impacts the structuring of the archaeal community (Tripathi et al., 2015; Zhou et al., 2017). Some lineages of Euryarchaeota are alkaline-tolerant and usually dominant in saline environments (Jones et al., 1998). Nevertheless, many other archaea are vulnerable to alkaline environments. ANMEs, especially the three species (i.e., ACR1, ACR32, and ACR111) from ANME-1b and ANME-2b (Figure 3A), dominate the archaeal community in all aggregates. They may be the fast-evolving species under certain environmental selection pressures (i.e., methane and pH) to defend their niches as the fast-running Red Queen in *Alice's Adventures in Wonderland* (Van Valen, 1977). Numerous studies have reported that some lineages of ANME have an alkaline preference and have high AOM activity in environments of higher pH (Nauhaus et al., 2005; Yao et al., 2023). Hence, these ANME species seem to win the “arms race” and occupy more niches, implying that the decrease in archaeal diversity may be due to the loss of niches caused by pH variation and the fast growth of the dominant species.

However, it is unsurprising that the composition of bacterial communities was impacted by sulfate, as it was dominated by SRB and SOB. However, its alpha diversity exhibited a hump-shaped transition and peaked at MB.R. As indicated by the geochemical variables, MB.R is potentially a transitional phase, in that the species in CB and MV co-exist (Figure 3B, Supplementary Figures 3–5). Microorganisms inhabiting here may benefit from resources, for example, sulfate and oxygen from seawater, as well as methane, sulfide, and metabolites (e.g., acetate; Yang et al., 2020), from the SMTZ, decreasing niche selection by reducing resource competition and causing an increase in diversity in MB.R (Brown, 1981; Fuhrman et al., 2008).

Although the exact interaction cannot be revealed by any correlation-based analysis, the network associations may indicate potential interspecies interactions to some extent (Fuhrman, 2009; Berry and Widder, 2014). A tight association between network structure and silicate indicates that pH could be the only factor modulating the interspecies associations (Figure 2C). An increase in the overall negative associations reflects more potential interspecies co-exclusion from NS to MV (Fuhrman, 2009; Berry and Widder, 2014). Reduction or depletion of sulfate in MB.B and MV may cause a shortage of electron receptors for AOM, which probably induces interspecies competition among ANME and SRB (Supplementary Figure 4) for limiting resources (Boetius and Wenzhöfer, 2013). Hence, it is probably the sulfate limitation that causes the interspecies co-exclusion. However, it is worth noticing that sulfate depletion is attributed to microbial metabolism rather than the fluids themselves (Joye, 2020). Methane is the most important carbon and energy source for cold-seep ecosystems (Suess, 2020). In marine sediments, more than 80% of the emitted methane is converted via AOM into carbonates (Hinrichs and Boetius, 2002). Although many other electron receptors are identified to be involved in AOM (Beal et al., 2009; Ettwig et al., 2010), sulfate is the most accessible one in a marine environment (Boetius and Wenzhöfer, 2013). Moreover, in CB, the oxidation of hydrocarbons by SRB via the reverse Wood-Ljungdahl and β-oxidation pathways coupled with sulfate reduction (Bombach et al., 2009; Kleindienst et al., 2014; Abu Laban et al., 2015; Kleindienst and Knittel, 2020) could be responsible for the sulfide production. However, the highly abundant JS-1 clade may play a role in the fermentation of non-methane hydrocarbons into fatty acids for further biosynthesis rather than carbonates (Liu et al., 2019). Conversely, carbonate and sulfide can be removed by biological and chemical reactions, drawing back the alkalization. Calcium reacts with carbonate to form authigenic carbonates, which are by far the most widely encountered footprint of cold seeps (Suess, 2020).

Moreover, the carbon fixation and sulfide oxidation metabolisms mediated by the autotrophs also result in the consumption of carbonate and sulfide. It is interesting that *Sulfurovum*, a chemolithoautotrophic SOB, is ubiquitous in CB, MB, and MV. They can couple the oxidation of reductive sulfur compounds with the fixation of inorganic carbonate, playing an essential role in carbon and sulfur cycling (Assié et al., 2020). This clade is well-known for fixing carbon dioxide via the rTCA cycle, but recent studies have revealed that several lineages of it gain the CBB cycle via horizontal acquisition (Assié et al., 2020). The highly abundant Milano-WF1B-44 in MV was first identified as a symbiotic autotrophic sulfide oxidizer that is affiliated with Gammaproteobacteria (Eisen et al., 1992; Krueger et al., 1996; Russell et al., 2017). Several members of Gammaproteobacteria are capable of fixing carbon via both rTCA and CBB cycles (Rubin-Blum et al., 2019). We noticed the alternation of carbon fixation mechanisms between different aggregates (Figures 5, 6), but more clues are warranted for precise clarification of the carbon fixer in each aggregate. Taken together, sulfate reduction coupled with hydrocarbon oxidation and sulfide oxidation-coupled carbon fixation may maintain the dynamic balance of pH. However, increasing methane flux may tip the balance in one direction and subsequently drive alkalization and microbial community alteration in sediment.

Previous studies have found that the SRB usually settles down prior to the SOB (especially *Sulfurovum*) and methanotrophs after the seepage begins (Ruff et al., 2019; Thurber et al., 2020), indicating that the metabolisms of the SRB may create new niches for the SOB (especially *Sulfurovum*) and methanotrophs. Although such a phenomenon was not observed in this study, our metagenomic results may shed light on the underlying mechanisms. A high sulfide concentration in CB, inferred by the occurrence of the sulfide-dependent Vesicomyidae clam aggregates inhabited (Kleindienst, 2012), is potentially a result of highly intense DSR metabolism mediated by SRB but less intense sulfide oxidation inferred by the low abundance of *sqr* and *fccB* genes (Figure 5). Therefore, environmental alternations made by SRB may create a niche for SOB, which is in accordance with the niche construction theory (Laland et al., 2016). We characterize SRB and SOB as the key functional taxa in microbial community structuring. Both of them directly participate in the carbon and sulfur cycles, dynamically balancing the biogeochemical cycles and potentially impacting the environment.

## Conclusion

The methane flux fundamentally drives changes in the environment (i.e., pH and sulfur compounds) that correlate with distinct microbial communities. Keystone functional lineages related to geochemical cycling, including ANME, SRB, and so on, showed clear aggregate-specific distribution patterns. These results have shown that the heterogeneity of the environment shaped the C and S cycling-associated microbial community in different faunal aggregates in the cold seep. Furthermore, the hydrocarbon oxidation-coupled sulfate reduction and sulfur compound oxidation-coupled carbon fixation mediated by ANMEs, SRB, and SOB likely dynamically balance the carbon and sulfur cycles that significantly impact pH and sulfur compound supplement, which may shape the microbial community in turn (Figure 6).

## Data availability statement

The datasets presented in this study can be found in online repositories. The names of the repository/repositories and accession number(s) can be found in the article/Supplementary material.

## Author contributions

YC, JL, and SZ conceived the original concept. YC, QL, PD, and NL collected the samples. NL, YL, and LL analyzed the geochemical variables. Data analyses were performed by YC and TD. The manuscript was written by YC, TD, and JL. All authors contributed to the article and approved the submitted version.
