# Supplementary material for: Environmental heterogeneity shapes the C and S cycling-associated microbial community in Haima's cold seeps
Source: Front Microbiol. 2023 Jul 4;14:1199853. doi: 10.3389/fmicb.2023.1199853 (PMC10370420; doi:10.3389/fmicb.2023.1199853)
Supplement: Supplementary file 1 [file Data_Sheet_1.pdf]

Supplementary data for

Environmental heterogeneity shapes C and S cycling associated microbial community in Haima cold seep

Yu Chen[1]†, Tianjiao Dai[3]†, Niu Li[2], Qiqi Li[2], Yuanjiao Lyu[2], Pengfei Di[2], Lina Lyu[2], Si Zhang[1,2*], Jie Li[2*]

[1] Southern Marine Science and Engineering Guangdong Laboratory (Guangzhou), China, 511458

[2] CAS Key Laboratory of Tropical Marine Bio-resources and Ecology, South China Sea Institute of Oceanology, Chinese Academy of Sciences, Guangzhou, Guangdong, China

[3]School of Water Resources and Environment, China University of Geosciences (Beijing), Beijing, China

†These authors contributed equally: Yu Chen, Tianjiao Dai

*Corresponding:

Jie Li, lijietaren@scsio.ac.cn;

Si Zhang, zhsimd@scsio.ac.cn

Supplementary Table 1. Samples applied for corresponding analyses in this study.

Pushcore	Sample	Depth (cm)	16S rRNA amplicon		Metagenome	Group
			Bacteria	Archaea		
HM-S1	HM-S1-1	0-3	√	√	√	NS
	HM-S1-2	3-6	√	√	√	NS
	HM-S1-3	6-9	√		√	NS
	HM-S1-4	9-12	√		√	NS
	HM-S1-5	12-15	√	√	√	NS
	HM-S1-6	15-18	√	√	√	NS
	HM-S1-7	18-21	√	√	√	NS
	HM-S1-8	21-24	√	√	√	NS
HM-S2	HM-S2-4	9-12	√	√	√	CB
	HM-S2-5	12-15	√	√	√	CB
	HM-S2-6	15-18	√	√	√	CB
	HM-S2-7	18-21	√	√	√	CB
	HM-S2-8	21-24	√	√		CB
	HM-S2-9	24-27	√	√		CB
	HM-S2-10	27-30	√	√		CB
	HM-S2-11	30-33	√	√	√	CB
	HM-S2-12	33-36	√	√	√	CB
HM-S3A	HM-S3A-1	0-3	√	√	√	MB.R
	HM-S3A-2	3-6	√	√	√	MB.R
	HM-S3A-3	6-9	√	√		MB.R

	HM-S3A-4	9-12	√	√		MB.R
	HM-S3A-5	12-15	√	√		MB.B
	HM-S3A-6	15-18	√	√		MB.B
	HM-S3A-7	18-21	√	√		MB.B
	HM-S3A-8	21-24	√	√		MB.B
HM-S3B	HM-S3B-2	3-6	√	√		MB.R
	HM-S3B-3	6-9	√	√		MB.R
	HM-S3B-4	9-12	√	√		MB.R
	HM-S3B-5	12-15	√	√	√	MB.R
	HM-S3B-6	15-18	√	√		MB.R
	HM-S3B-7	18-21	√	√		MB.B
	HM-S3B-8	21-24	√	√		MB.B
HM-S4	HM-S4-1	0-3	√	√	√	MV
	HM-S4-2	3-6	√	√	√	MV
	HM-S4-3	6-9	√	√	√	MV
	HM-S4-4	9-12	√	√	√	MV
	HM-S4-5	12-15	√	√	√	MV
	HM-S4-6	15-18	√	√	√	MV
	HM-S4-7	18-21	√	√	√	MV
	HM-S4-8	21-24	√	√	√	MV

Supplementary Table 2. ANOSIM based on Euclidean dissimilarity distance for geochemical variables.

Group	ANOSIM	
	R	*p*
NS vs. CB	0.87	0.001
NS vs. MB.R	0.65	0.001
NS vs. MB.B	0.75	0.002
NS vs. MV	1.00	0.001
CB vs. MB.R	0.39	0.001
CB vs. MB.B	0.70	0.001
CB vs. MV	1.00	0.002
MB.R vs. MB.B	0.10	0.14
MB.R vs. MV	0.94	0.001
MB.B vs. MV	0.64	0.002

Supplementary Table 3. ANOSIM and PERMANOVA based on Bray-Curtis dissimilarity distance for archaeal communities.

Group	ANOSIM		PERMANOVA	
	R	*p*	R^2	*p*
NS vs. CB	0.80	0.001	0.73	0.001
NS vs. MB.B	0.77	0.002	0.71	0.001
NS vs. MB.R	0.98	0.001	0.73	0.001
NS vs. MV	1.00	0.001	0.54	0.001
CB vs. MB.B	0.43	0.004	0.66	0.001
CB vs. MB.R	0.99	0.002	0.68	0.002
CB vs. MV	1.00	0.002	0.42	0.001
MB.R vs. MB.B	0.95	0.007	0.65	0.001
MV vs. MB.B	0.99	0.001	0.45	0.001
MV vs. MB.R	0.99	0.001	0.51	0.001

Supplementary Table 4. ANOSIM and PERMANOVA analyses based on Bray-Curtis dissimilarity distance for bacterial communities.

Group	ANOSIM		PERMANOVA	
	R	*p*	R^2	*p*
NS vs. CB	1.00	0.001	0.51	0.001
NS vs. MB.B	1.00	0.002	0.65	0.001
NS vs. MB.R	1.00	0.001	0.68	0.001
NS vs. MV	1.00	0.001	0.73	0.002
CB vs. MB.B	0.80	0.001	0.45	0.001
CB vs. MB.R	0.64	0.001	0.42	0.001
CB vs. MV	0.82	0.001	0.54	0.001
MB.R vs. MB.B	0.98	0.002	0.66	0.001
MV vs. MB.B	1.00	0.002	0.71	0.001
MV vs. MB.R	1.00	0.001	0.73	0.001

Supplementary Table 5. Spearman correlation between archaeal alpha diversity indices and geochemical variables.

Alpha diversity indices	Chloride		Sulfate		Magnesium		Calcium		Ammonium		Nitrite		Nitrate		Silicate		Phosphate	
	R	*p*	R	*p*	R	*p*	R	*p*	R	*p*	R	*p*	R	*p*	R	*p*	R	*p*
Richness	-0.01	0.98	0.67	**<0.001**	0.25	0.13	0.59	**<0.001**	-0.64	**<0.001**	-0.45	**0.01**	-0.21	0.21	-0.52	**<0.001**	-0.56	**<0.001**
Shannon	0.22	0.2	0.75	**<0.001**	0.23	0.17	0.60	**<0.001**	-0.60	**<0.001**	-0.19	0.27	-0.05	0.79	-0.73	**<0.001**	-0.56	**<0.001**
Simpson	0.41	**0.01**	0.65	**<0.001**	0.19	0.25	0.49	**<0.001**	-0.44	**0.01**	0.01	0.96	0.07	0.68	-0.68	**<0.001**	-0.42	**0.01**
Pielou	0.31	0.06	0.68	**<0.001**	0.13	0.43	0.48	**<0.001**	-0.44	**0.01**	-0.01	0.97	0.00	0.99	-0.79	**<0.001**	-0.50	**<0.001**
Chao1	-0.01	0.98	0.67	**<0.001**	0.25	0.13	0.59	**<0.001**	-0.64	**<0.001**	-0.45	**0.01**	-0.21	0.21	-0.52	**<0.001**	-0.56	**<0.001**
ACE	0.00	0.98	0.66	**<0.001**	0.27	0.11	0.58	**<0.001**	-0.62	**<0.001**	-0.45	**0.01**	-0.22	0.2	-0.52	**<0.001**	-0.59	**<0.001**
GoodsCov	-0.04	0.81	-0.63	**<0.001**	-0.46	**<0.001**	-0.60	**<0.001**	0.69	**<0.001**	0.47	**<0.001**	0.13	0.45	0.17	0.31	0.40	**0.01**

Supplementary Table 6. Spearman correlation between bacterial alpha diversity indices and geochemical variables.

Alpha diversity indices	Chloride		Sulfate		Magnesium		Calcium		Ammonium		Nitrite		Nitrate		Silicate		Phosphate	
	R	*p*	R	*p*	R	*p*	R	*p*	R	*p*	R	*p*	R	*p*	R	*p*	R	*p*
Richness	-0.16	0.33	0.04	0.83	0.18	0.26	0.06	0.71	<0.01	0.99	-0.49	**<0.001**	-0.45	**<0.001**	-0.23	0.15	-0.20	0.21
Shannon	-0.20	0.22	0.04	0.81	0.11	0.48	0.07	0.65	0.01	0.97	-0.45	**<0.001**	-0.55	**<0.001**	-0.17	0.3	-0.20	0.22
Simpson	-0.19	0.24	0.08	0.63	0.08	0.61	0.09	0.56	-0.03	0.85	-0.42	**0.01**	-0.53	**<0.001**	-0.20	0.22	-0.20	0.22
Pielou	-0.16	0.32	0.09	0.6	0.05	0.74	0.13	0.42	-0.03	0.86	-0.22	0.17	-0.52	**<0.001**	-0.06	0.7	-0.16	0.31
Chao1	-0.15	0.36	0.05	0.75	0.20	0.22	0.08	0.61	-0.02	0.89	-0.49	**<0.001**	-0.42	**0.01**	-0.24	0.13	-0.22	0.18
ACE	-0.15	0.35	0.04	0.8	0.19	0.25	0.07	0.68	-0.01	0.95	-0.48	**<0.001**	-0.42	**0.01**	-0.24	0.13	-0.21	0.19
GoodsCov	-0.12	0.44	0.19	0.25	-0.02	0.88	0.21	0.2	-0.18	0.26	0.16	0.31	0.001	0.86	0.041	0.21	<0.001	0.91

Supplementary Table 7. Topological indices of MESs for microbial community associations in different succession stages.

Topological indices	NS	CB	MB.R	MB.B	MV
Coefficient cutoff	0.57	0.91	0.79	0.45	0.32
Total nodes	74	93	150	55	92
Total links	835	300	1141	774	2203
Negative links (%)	40.8	4.0	8.1	43.4	52.8
R^2 of power-law	0.008	0.695	0.41	0.035	0.221
Average degree (avgK)	22.568	6.452	15.213	28.145	47.891
Average clustering coefficient (avgCC)	0.691<br>0.479 +/- 0.010[a]	0.374<br>0.148 +/- 0.017[a]	0.426<br>0.358 +/- 0.014[a]	0.680<br>0.601 +/- 0.005[a]	0.635<br>0.588 +/- 0.002[a]
Average path distance (GD)	1.83<br>1.730 +/- 0.006[a]	4.086<br>2.638 +/- 0.049[a]	3.777<br>2.367 +/- 0.028[a]	1.479<br>1.481 +/- 0.001[a]	1.474<br>1.474 +/- 0.000[a]
Geodesic efficiency (E)	0.632<br>0.648 +/- 0.001[a]	0.339<br>0.431 +/- 0.005[a]	0.372<br>0.478 +/- 0.004[a]	0.76<br>0.760 +/- 0.000[a]	0.763<br>0.763 +/- 0.000[a]
Harmonic geodesic distance (HD)	1.582<br>1.543 +/- 0.002[a]	2.947<br>2.320 +/- 0.029[a]	2.689<br>2.091 +/- 0.017[a]	1.315<br>1.315 +/- 0.000[a]	1.31<br>1.310 +/- 0.000[a]
Centralization of betweenness (CB)	0.069<br>0.043 +/- 0.008[a]	0.404<br>0.140 +/- 0.018[a]	0.093<br>0.062 +/- 0.008[a]	0.016<br>0.016 +/- 0.003[a]	0.01<br>0.007 +/- 0.001[a]
Centralization of stress centrality (CS)	0.598<br>0.282 +/- 0.037[a]	4.349<br>0.513 +/- 0.052[a]	2.072<br>0.394 +/- 0.044[a]	0.131<br>0.147 +/- 0.011[a]	0.15<br>0.125 +/- 0.008[a]
Centralization of eigenvector centrality (CE)	0.11<br>0.108 +/- 0.003[a]	0.248<br>0.239 +/- 0.015[a]	0.14<br>0.135 +/- 0.003[a]	0.07<br>0.065 +/- 0.002[a]	0.046<br>0.040 +/- 0.001[a]
Density (D)	0.309<br>0.309 +/- 0.000[a]	0.07<br>0.070 +/- 0.000[a]	0.102<br>0.102 +/- 0.000[a]	0.521<br>0.521 +/- 0.000[a]	0.526<br>0.526 +/- 0.000[a]

Efficiency	0.7<br>0.700 +/- 0.000 [a]	0.929<br>0.939 +/- 0.002 [a]	0.886<br>0.902 +/- 0.002 [a]	0.488<br>0.488 +/- 0.000 [a]	0.479<br>0.479 +/- 0.000 [a]
Modularity	0.256<br>0.074 +/- 0.017 [a]	0.446<br>0.196 +/- 0.022 [a]	0.206<br>0.050 +/- 0.006 [a]	0.137<br>0.064 +/- 0.018 [a]	0.142<br>0.056 +/- 0.010 [a]

[a]Topological indices (mean ±sd) of 100 random networks generated by randomly rewiring the corresponding empirical network.

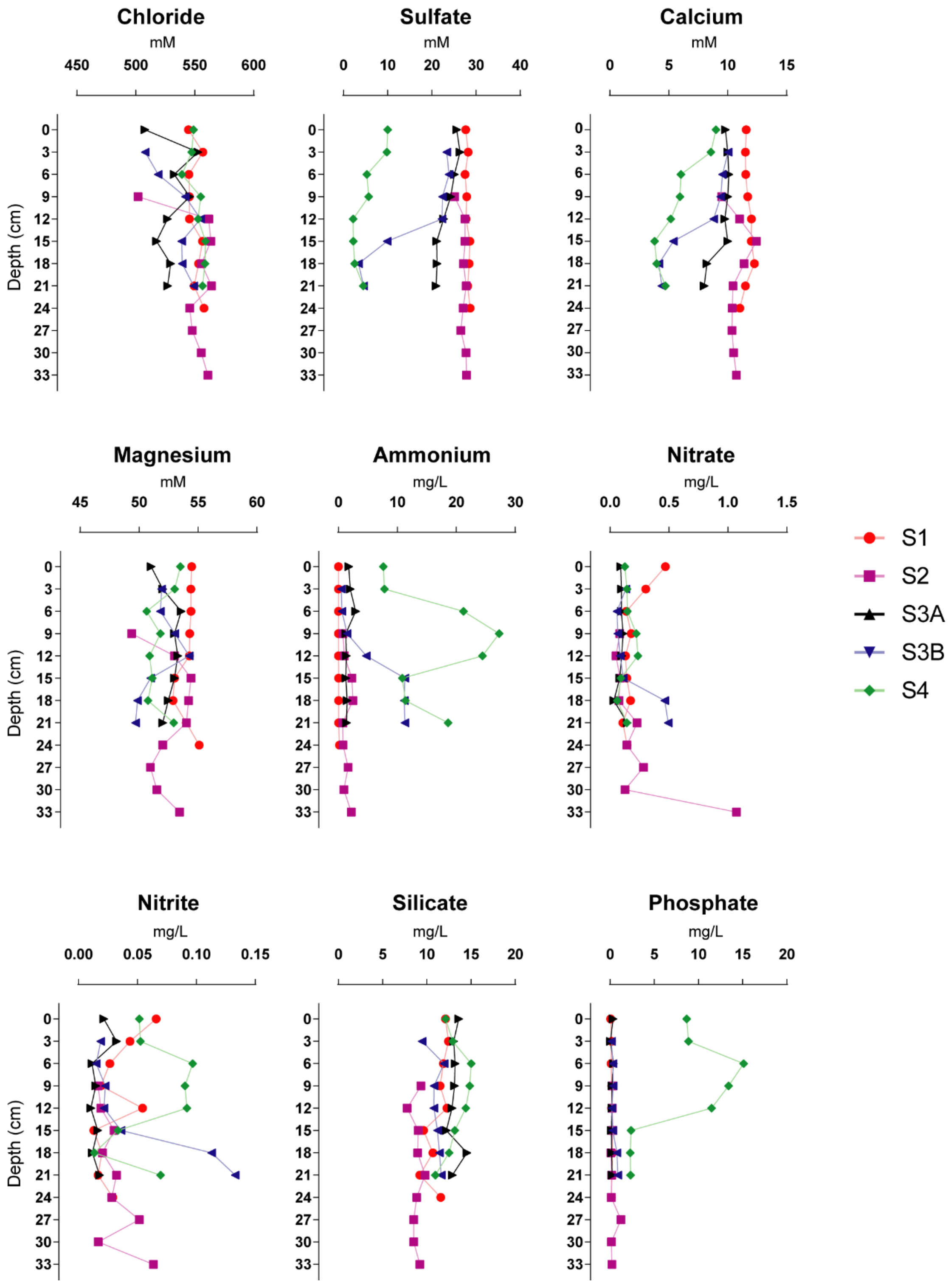

Supplementary Figure 1. Geochemical profiling of pushcores used in this study. Nine geochemical variables, including chloride, sulfate, calcium, magnesium, ammonium, nitrate, nitrite, silicate, and phosphate, were examined for each pushcore.

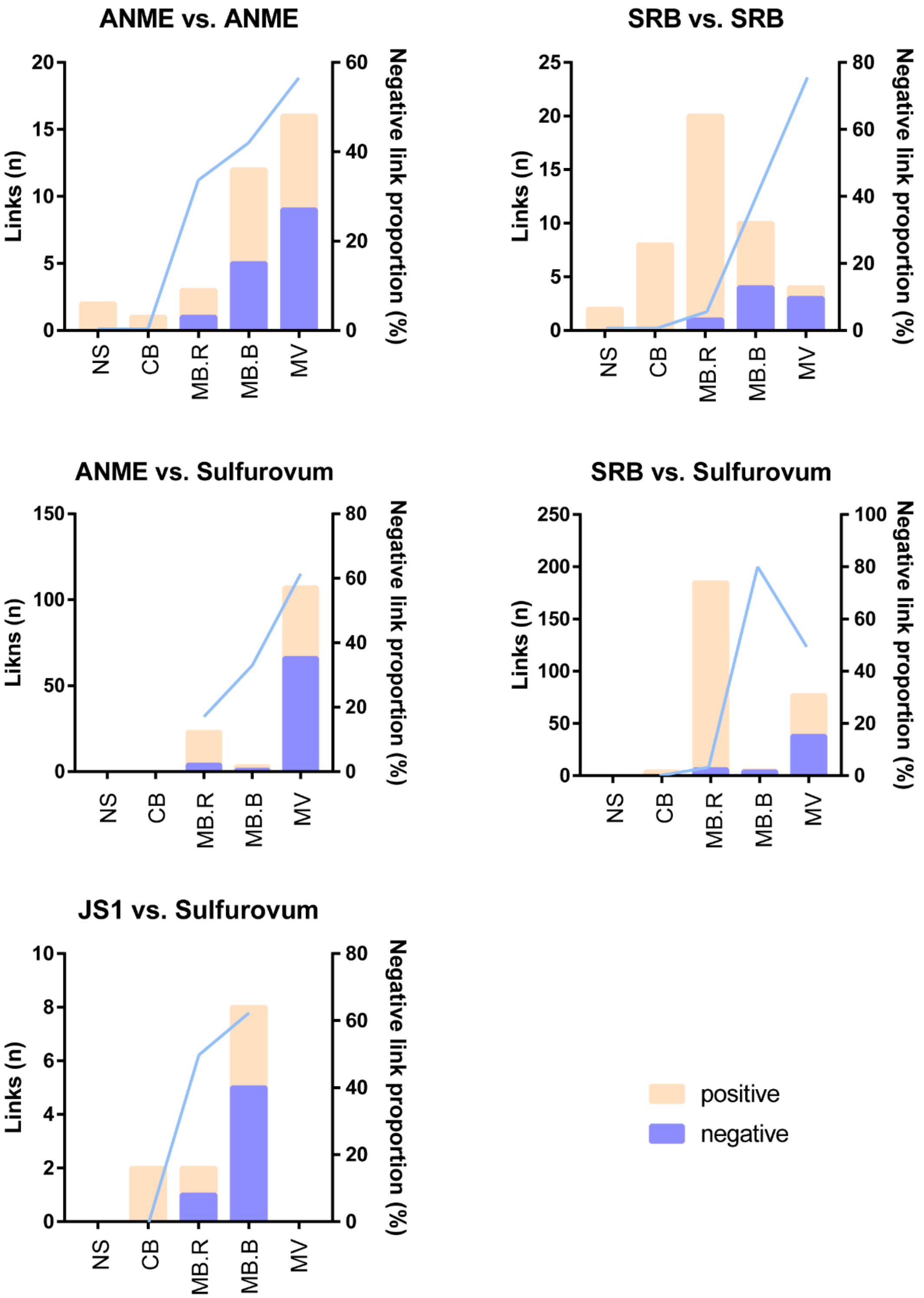

Supplementary Figure 2. Positive and negative links within and between the networked anaerobic methanotrophic archaea (ANME), sulfate reducing bacteria (SRB), JS-1, and *Sulfurovum* species for each stages. The bars indicated the number of positive/negative links, and the blue lines indicated the proportion of negative links.

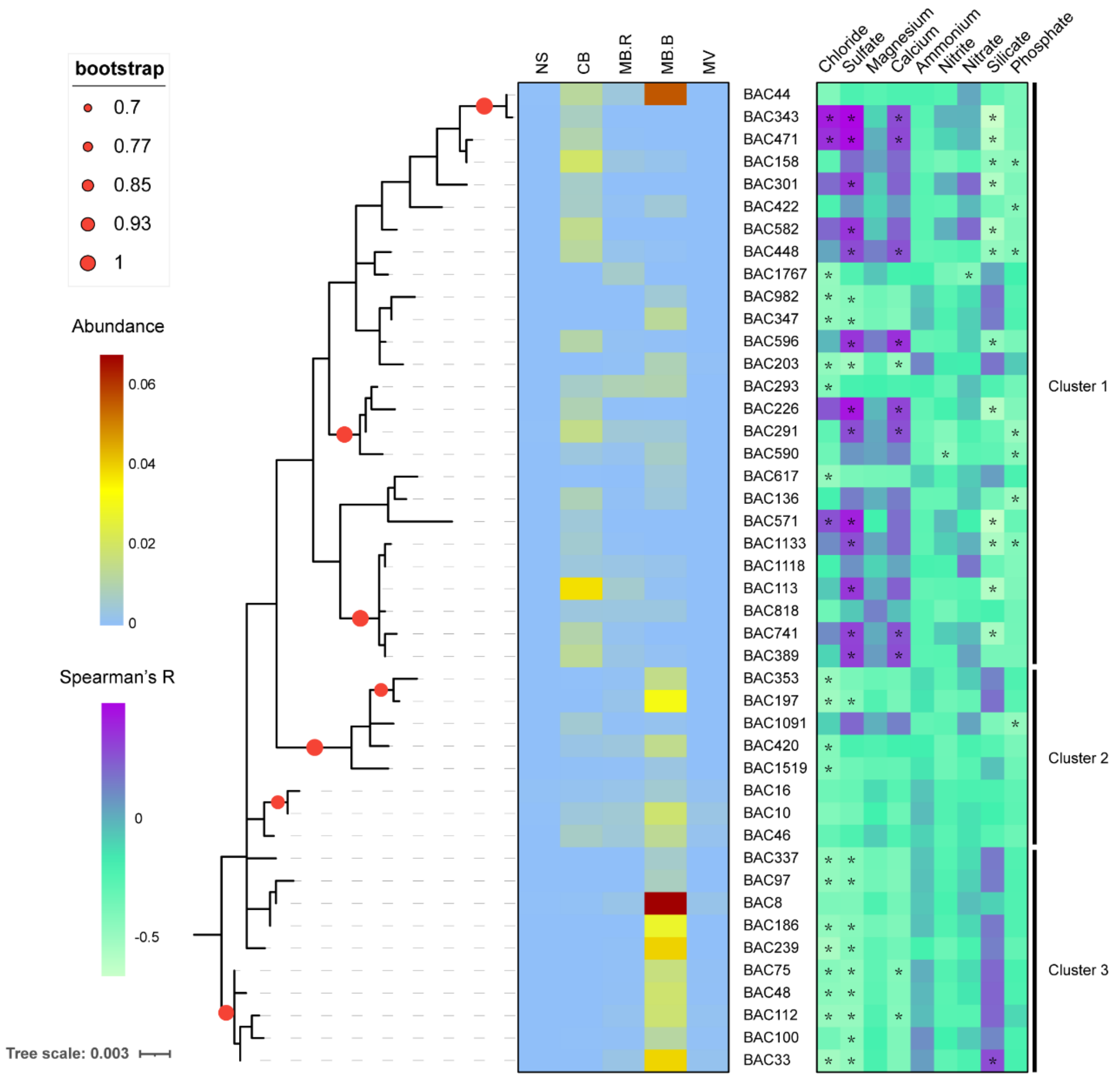

Supplementary Figure 3. Phylogenic relationship and distribution pattern of JS-1. Maximum Likelihood phylogenic trees were constructed for networked JS-1 ASVs with 16S rRNA gene sequences. Sequences from Actinobacteria were selected as outgroup. Asterisks indicated significant correlations between ASV abundance and geochemical variables (Spearman, |R| > 0.3, $p < 0.05$).

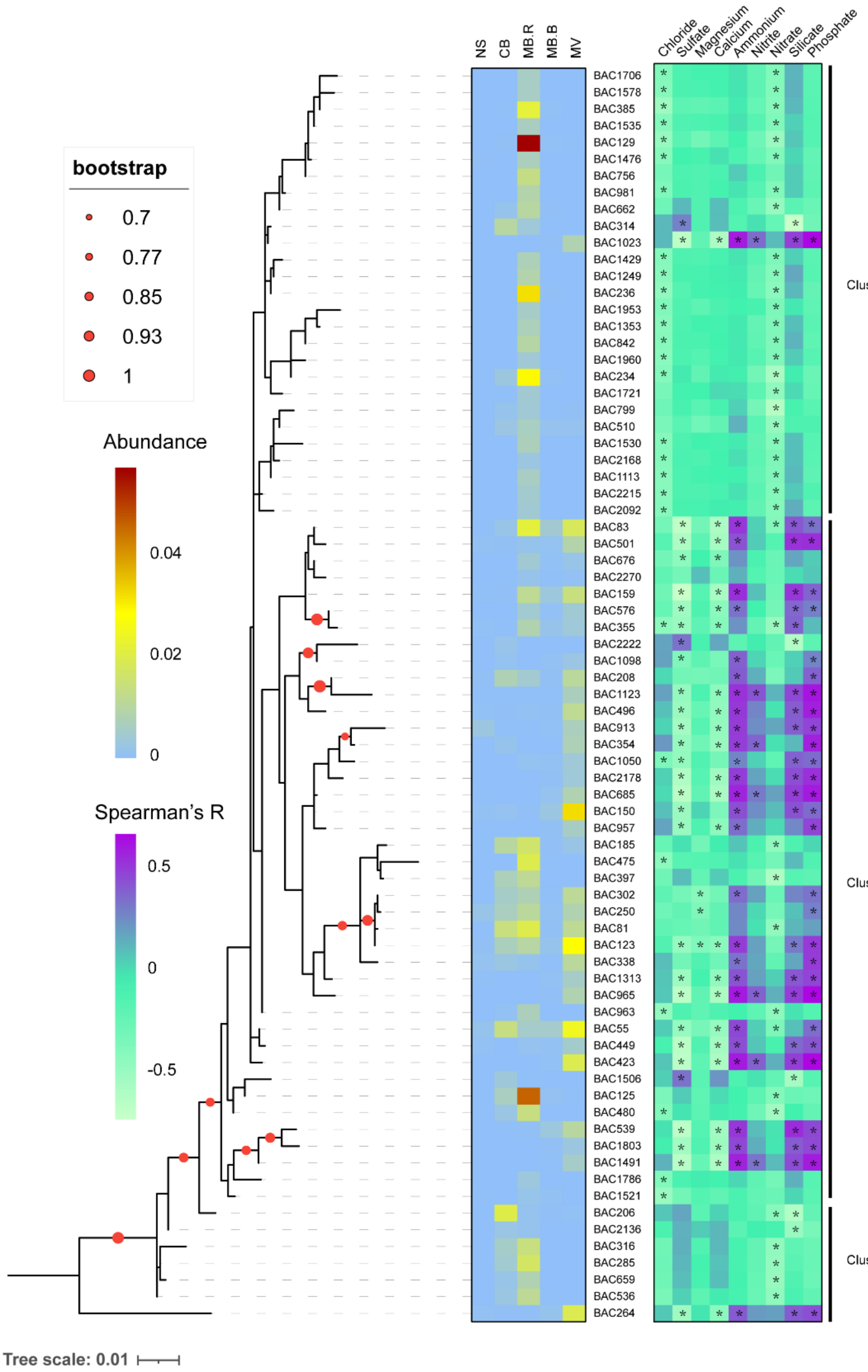

Supplementary Figure 4. Phylogenic relationship and distribution pattern of *Sulfurovum*.

Maximum Likelihood phylogenic trees were constructed for networked *Sulfurovum* ASVs with 16S rRNA gene sequences. Sequences from Actinobacteria were selected as outgroup. Asterisks indicated significant correlations between ASV abundance and geochemical variables (Spearman, $|R| > 0.3$, $p < 0.05$).

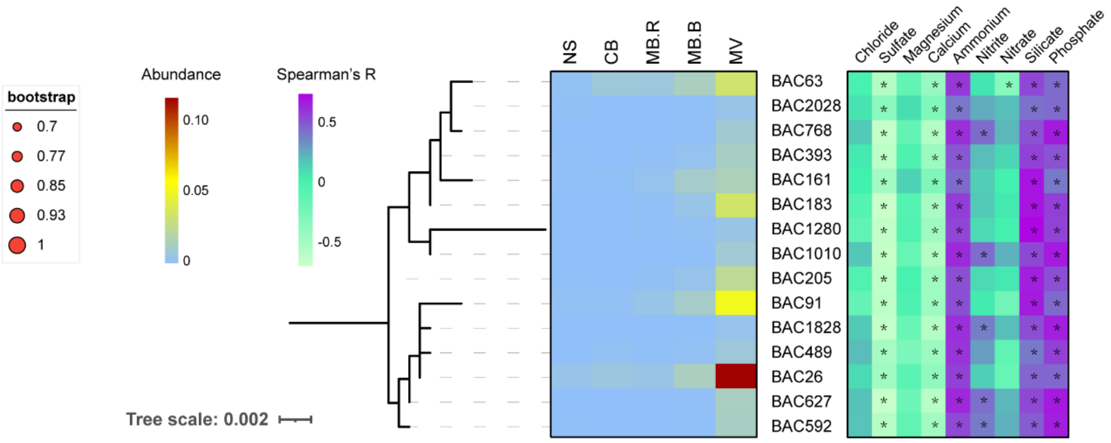

Supplementary Figure 5. Phylogenic relationship and distribution pattern of Milano-WF1B-44. Maximum Likelihood phylogenic trees were constructed for networked Milano-WF1B-44 ASVs with 16S rRNA gene sequences. Sequences from Actinobacteria were selected as outgroup. Asterisks indicated significant correlations between ASV abundance and geochemical variables (Spearman, $|R| > 0.3$, $p < 0.05$).
